# Troxerutin protect sperm, seminiferous epithelium and pituitary-gonadal axis from torsion-detorsion injury: An experimental study

**Published:** 2018-05

**Authors:** Abdolreza Kheirollahi, Abolfazl Abbaszadeh, Khatereh Anbari, Behrouz Rostami, Amirhossein Ahangari, Afshin Hasanvand, Mohammadreza Gholami

**Affiliations:** 1 *Razi Herbal Medicines Research Center, Lorestan University of Medical Sciences, Khorramabad, Iran.*; 2 *Department of Surgery, Faculty of Medicine, Lorestan University of Medical Sciences, Khorramabad, Iran.*; 3 *Social Determinant of Health Research Center, Faculty of Medicine, Lorestan University of Medical Sciences, Khorramabad, Iran.*; 4 *Student Research Committee, Lorestan University of Medical Sciences, Khorramabad, Iran.*; 5 *Department of Anatomical Sciences, Faculty of Medicine, Kermanshah University of Medical Sciences, Kermanshah, Iran.*

**Keywords:** Troxerutin, Torsion, Testis, Spermatogenesis, Spermatozoa

## Abstract

**Background:**

Troxerutin is a flavonoid antioxidant that protect different organ against damage caused by ischemia-reperfusion**.**

**Objective:**

The aim of this study was to evaluate the effect of troxerutin in reducing the damages caused by ischemia-reperfusion in rat's testis.

**Materials and Methods:**

40 Male Wistar rats (2 month old) were divide to four groups (n=10). Group1 (sham), Group 2 (control, ischemia-reperfusion (I/R) without treatment), Group 3 (I/R+150 mg/kg of troxerutin), and group 4 (I/R+20 mg/kg of vitamin C). Treatment of group 3 and group 4 during torsion (twists 720 counter clock at 90 min) followed by 50 days detorsion. After 50 days, blood samples were collected and rats in all study groups were killed and their testes were removed, and fixed with Bouin’s solution. Testis was stained with hematoxylin and eosin dye and the level of testosterone, luteinizing hormone (LH) and follicle-stimulating hormone (FSH) were measured with ELISEA methods. TUNEL was employed to detect apoptosis. Epididymis caudal part was removed and total sperm count was determined. Johnson techniques were used for assessment of seminiferous tubules quality.

**Results:**

Troxerutin treated group has higher Johnson score’s (p≤0.001), antiapoptotic properties (p≤0.001), sperm count (p=0.065), and higher LH (p≤0.001), FSH (p≤0.001) and testosterone (p=0.002) levels than control group. Vitamin C treated group showed increase level of testosterone but didn’t show significant differences on the number of apoptotic cells, Johnson scores, LH, FSH and sperm count than control group.

**Conclusion:**

Troxerutin has protective effects on testicular torsion induced injury and can ameliorate spermatogenesis in the torsion-detorsion models.

## Introduction

Testicular torsion is an acute surgical emergency that the delay in its treatment leads to ischemia and testicular injury (1). After interrupting the arterial blood flow, testis apoptosis will develope in a few hours (2-4). Testicular torsion is a serious problem in men, that if not treated can be lead to reduce fertility (3). There are many other suggested etiological or contributory factors which show the serious roles of oxidative stress in male infertility. These factors should be considered in examining the causes of male infertility and stages of their treatment (3). Studies have reported high levels of free radicals in 25-40% of infertile men (5-10). The primary pathological mechanism of testicular damage is not fully understood, but free oxygen radicals formed during the ischemia-reperfusion have important role in this process (3, 11). Free oxygen radicals may cause the lipid peroxidation in cell membranes and mitochondria. Peroxidation of lipids causes the changes in membrane permeability and disruption in cell membrane integrity and the cell disorder finally (12-17). High concentrations of reactive oxygen species (ROS) have important role in pathophysiological damages of human spermatozoa’s (3). Oxidative stress has a major cause of infertility (3). Recent researches on oxidative stress and produced free oxygen radicals after testicular torsion show that antioxidants can protect testis against ROS damages (1, 3, 4, 11). Antioxidants play a key role in andrology (12, 13, 18-20). Antioxidants are substances that break down the chain oxidative reaction and reduce the oxidative stress (18, 19). Troxerutin belong to flavonoids family; that is including antioxidants compounds (21). This substance is derived from a natural bioflavonoid called rutin and normally comes from a tree called Sephora Japonica that grows in Japan (21). The main use of this substance is in the treatment of varicose veins as vasoprotective. This study display protective effects of troxerutin against testicles torsion-detorsion injury. 

The aim of this study was to evaluate the effect of troxerutin in reducing the damages caused by ischemia-reperfusion in rat testis.

## Materials and methods

**Animals and study design**

This experimental study was conducted at Lorestan University of Medical sciences. Experiments were performed on male Wistar rats, age 2 months, weighting 180-200 gr. In this study, 40 male rats were divided into four groups (n=10/each). All rats were kept in good conditions with 12 hr light/dark cycle, control temperature of 22±2^o^C, and free access to food and water. Since the welfare of animals used in this research was very important, every effort was made to reduce the suffering and the number of animals.

Group 1 or sham group: This group doesn’t receive treatment and surgery.

Group 2 or control group: This group was under the torsion followed by 50 days detorsion.

Group 3 or experimental 1: Treatment with troxerutin (150 mg/kg, gavage) during torsion followed by 50 days detorsion.

Group 4 or experimental 2: Treatment with vitamin C (20 mg/kg, gavage) during torsion followed by 50 days detorsion.

**Torsion induction**

The rats were anesthetized intraperitoneally with ketamine HCl (50 mg/kg) and xylazine (5 mg/kg) in accordance with the protocol approved by the Animal Care and Use Committee. Testicular torsion-detorsion applied with twists 720 counter clock at 90 min. In the experimental and control groups torsion followed by 50 days detorsion and treatment done according to the study design. After completion of treatment, blood samples were collected and then all animals were killed by overdose of anesthesia and their testes were removed from the abdominal cavity.

**Histopathological studies**

Testes were fixed in Bouin’s solution and embedded in paraffin, then they were cut, dehydrated and stained with hematoxylin-eosin. About 30 rounds or nearly round cross-sections of seminiferous tubules at same stage (stage 8) were randomly chosen in each rat. The seminiferous tubules were rated for their modified spermatogenesis index (SI) by Johnson’s score on a scale of 0-10 according to the range from no cells to complete spermatogenesis (22).

**Sperm analysis**

Sperm count and motility was evaluated by light microscopy at a magnification of 400× and non-progressive motility and immotility of spermatozoa were reported as percentage. In order to sperm motility analysis, the cauda epididymis was cut and sperms were released in 5 ml of Ham's F-10 medium (Sigma, USA) containing 0.5% bovine serum albumin and incubation at 37^o^C (with 5% CO_2_) for 20 min. Then the cauda epididymis sperm reserves were determined and the total sperm count was determined using a hemocytometric method. Sperm motility was analyzed and reported as the mean of motile sperm according to the World Health Organization method. Sperm viability was evaluated by Eosin-Nigrosin staining test. Aniline blue staining was applied for morphology assessment. The slides were assessed for morphological abnormality in tail, neck or head.

**TUNEL assay**

TUNEL (TdT-mediated dUTP-X nick end labeling) kit (Roche Company, cat. No. 11684817910, USA) was employed to detect DNA breaks at the early stages of apoptosis. The number of apoptosis-positive cells was calculated in the seminiferous tubules of different groups of study. Testes were fixed in Bouin’s solution and embedded in paraffin, cut and dehydrated. After testis sections were dewaxed and rehydrated, they were pretreated with proteinase K, PCR (polymerase chain reaction) grade, for 15 min at room temperature. Finally testes sections were rinsed 3 times with phosphate buffer saline (PBS) and dried. Then 50 µl TUNEL reaction mixtures was added on sample, and incubated for 60 min at +37^o^C in a humidified chamber in the dark. Again testis sections were rinsed 3 times with PBS and dried. In the next step, 50 µl Convertor-POD was added on sample and testis sections were incubate in a humidified chamber for 30 min at 37^o^C. In the final step, 50-100 µl DAB substrate was added and slides were incubate for 10 min at 20^o^C, testis sections were rinsed 3 times with PBS. Samples were mounted and analyzed under light microscope.

**Serum follicle-stimulating hormone (FSH), luteinizing hormone (LH) and testosterone hormone measurement**

Blood samples were centrifuged, 5000 per min, and serum was separated from the blood. Total serum concentration of FSH, LH and testosterone was measured using ELISA. Each test was performed according to the manufactory method. 

**Ethical consideration**

The study protocol has been designed according to the ethical principles approved by international committees (LUMS.REC.1395. 224) and all experiments were performed in accordance with right of laboratory animal care of Lorestan University of Medical Sciences. Every effort made to reduce the suffering and the number of animals. 

**Statistical analysis**

Statistical analysis, with SPSS software (Statistical Package for the Social Sciences, version 22.0, SPSS Inc, Chicago, Illinois, USA), were performed using the Mann-Whitney U Test and Kruskal-Wallis test. p<0.05 was considered statistically significant. All data were expressed as mean±SD, mean of rank and sum of rank in each group.

## Results

**Histopathological studies**

**Figure 1:**
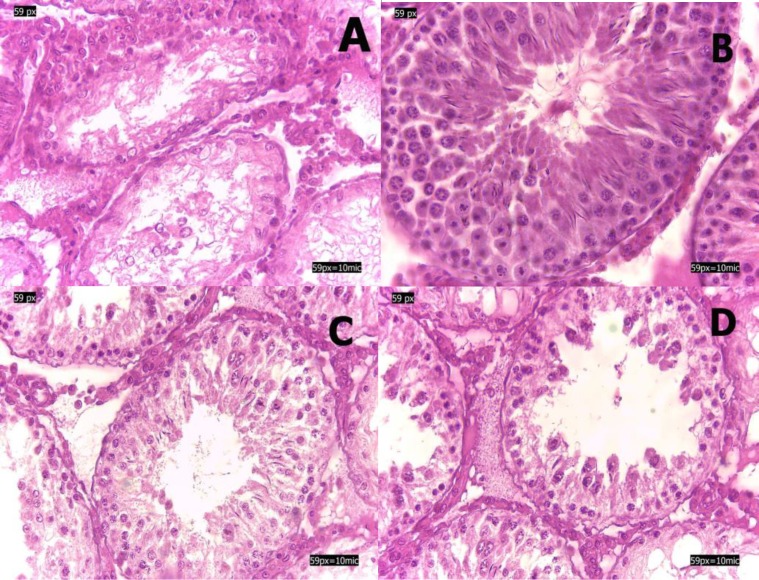
H and E staining of seminiferous tubules. A; control, B; sham, C; experimental 1, D; experimental 2. Section A show that seminiferous is destroyed and a few germ cells. Troxerutin administration, section C; experimental 1, preserve epithelium seminiferous from degeneration and germ cell, primary spermatocyte, secondary spermatocyte, and spermatid are obvious (Hematoxylin and Eosin, 400x).

In SI by Johnson’s score, an increase in the spermatogenic index was significant in the troxerutin treated group (p≤0.001). Therefore the SI shifted to high score during the treatment with troxerutin during ischemia. Treatment with vitamin C cannot significantly ameliorate the rate of Johnson score (p=0.878). The result shows the positive troxerutin impact on the improvement of histological changes of seminiferous tubules after testicular torsion (Figure 1). Analysing stained sections in the treated group with troxerutin compare to control show that troxerutin can preserve epithelium seminiferous from injury and degeneration (Figure 1).

**Sperm count**

The values of the mean sperm count as were significantly higher in troxerutin treated group when compared with other group (Table I). The results indicate administration of troxerutin improve sperm count in the comparison with control group (p=0.065). While troxerutin in the comparison with vitamin C doesn’t ameliorate sperm count (p=0.999). Treatment with vitamin C significantly ameliorate rate of sperm count in comparison with control (p=0.01). 

**Serum FSH, LH and testosterone hormone**

The results indicate administration of troxerutin improve FSH levels, LH levels (Table II), and testosterone levels (Table III) in comparison with control group. In addition, troxerutin in comparison with vitamin C ameliorate FSH, LH (Table II) and testosterone (Table III) levels. Treatment with vitamin C cannot significantly ameliorate the rate of FSH and LH (Table II) levels but levels of testosterone increased in the vitamin C treated in comparison with control. 

**Apoptosis analysis**

**Figure 2:**
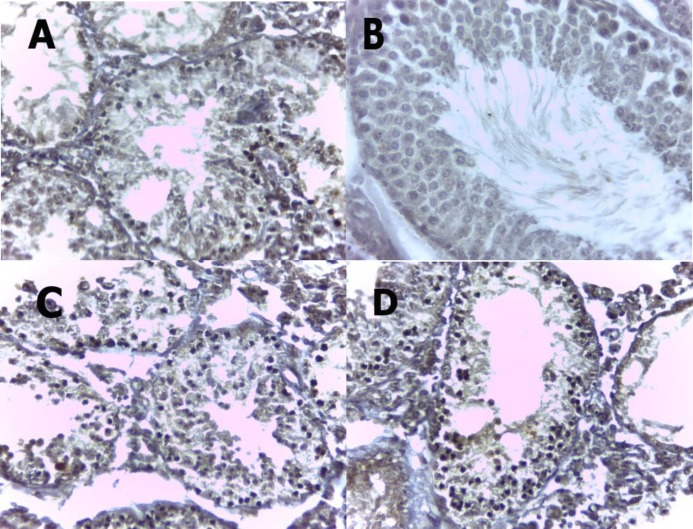
TUNEL assay of seminiferous tubules. A; control, B; sham, C; experimental 1, D; experimental 2. Cells stained dark brown are apoptotic cells while normal cells are stained blue (400X).

Evaluation of germ cell apoptosis showed that administration of troxerutin decreased number of apoptotic cells (Table III, Figure 2) in comparison with control group. Troxerutin in comparison with vitamin C decreased the number of apoptotic cells (Table III, Figure 2). Treatment with vitamin C cannot significantly decreased number of apoptotic cells (Table III, Figure 2) in comparison with control.

**Table I:** Data of seminiferous tubules Johnson̕ s scores and sperm count analysis

**Group of study**	**Seminiferous tubules Johnson score**				**Sperm count analysis**			
	**Sum of rank**	**Mean rank**	**Mean ** **±** ** SD**	**p-value**	**Sum of rank**	**Mean rank**	**Mean ** **±** ** SD**	**p-value**
Control	36	4.5	2.75 ± 0.46	≤0.001	50	6.25	13625 ±7026.77	0.065
Experimental 1	100	12.5	5.75 ± 0.07		86	10.75	18187.5 ± 7026.77	
Control	66	8.25	2.75 ±0.46	0.878	44	5.5	13625 ± 7026.77	0.01
Experimental 2	70	8.75	2.87 ±0.83		92	11.5	19000± 8035.6	
Experimental 1	100	12.5	5.75 ±0.07	≤0.001	68	8.5	18187.5 ±7026.77	0.999
Experimental 2	36	4	2.87 ± 0.83		68	8.5	19000± 8035.6	

Mann-Whitney U test

**Table II:** Data of LH and FSH assay were analyzed in different groups

**Group of study**	**LH assay analysis**				**FSH assay analysis**			
	**Sum of rank**	**Mean rank**	**Mean ** **±** ** SD**	**p-value**	**Sum of rank**	**Mean rank**	**Mean ** **±** ** SD**	**p-value**
Control	37.5	4.69	0.76 ±0.16	≤0.001	51	6.69	0.91± 0.15	0.083
Experimental 1	98.5	12.31	1.06 ±0.1		85	12.63	1.07 ± 0.1	
Control	36	4.5	0.76 ±0.16	0.878	69	8.63	0.91 ±0.15	0.959
Experimental 2	100	12.5	1.03± 0.05		67	8.38	1.92 ± 0.24	
Experimental 1	72.5	9.06	1.06 ±0.1	≤0.001	84	10.5	1.07 ±0.1	0.105
Experimental 2	63.5	7.94	1.03± 0.05		52	6.5	0.92 ±0.24	

Mann-Whitney U test.

LH: luteinizing hormone

FSH: follicle-stimulating hormone

**Table III:** Data of testosterone and TUNEL assay were analyzed in different groups

**Group of study**	**Testosterone assay analysis**				**TUNEL assay analysis**			
	**Sum of rank**	**Mean rank**	**Mean ** **±** ** SD**	**p-value**	**Sum of rank**	**Mean rank**	**Mean ** **±** ** SD**	**p-value**
Control	40	5	0.4±0.00	0.002	99	12.38	5.87±1.88	≤0.001
Experimental 1	96	12	0.75±0.2		37	4.63	1.5±1.19	
Control	36	4.5	0.4±0.00	≤0.001	91.5	11.44	5.87±1.88	0.01
Experimental 2	100	12.5	3.97±0.51		44.5	5.56	3.12±1.45	
Experimental 1	36	4.5	0.75±0.2	≤0.001	49	6.13	1.5±1.19	0.05
Experimental 2	100	12.5	3.97±0.51		87	10.88	3.12±1.45	

Mann-Whitney U test.

**Figure 3:**
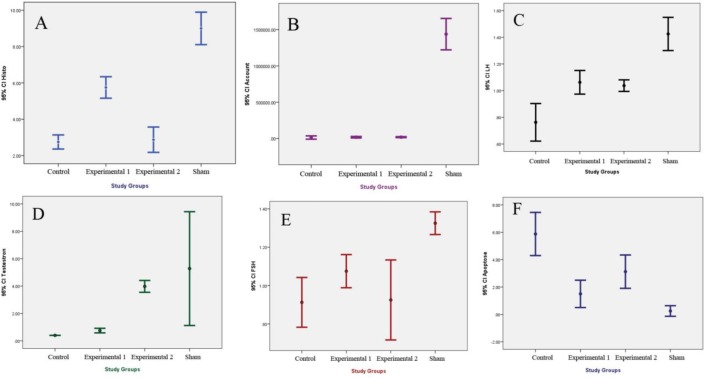
Comparing histological changes (A), sperm counts (B), LH assay (C), testosterone assay (D), FSH assay (E), and testis apoptosis (F) were performed in the study groups using the Kruskal-Wallis test

## Discussion

This study showed that troxerutin can ameliorate histopathological damage of testis and pituitary-gonadal axis hormone during testis IR. IR induced testis tissue damage with various mechanisms such as; increasing of ROS, production and release of inflammatory factors as well as the release of promoter apoptosis enzymes such as caspases (5, 19, 23). There is a lot of evidence that show the serious role of oxidative stress in male infertility, which should be considered in examining the causes of male infertility and stages of their treatment (5, 19, 23). Studies have reported high levels of free radicals in 25-40% of infertile men (20, 24, 25). Although the primary pathological mechanisms of testicular damage aren’t fully understood, but oxygen free radicals formed during the ischemia-reperfusion have important role in this process. Excessive amount of oxygen free radicals cause lipid peroxidation in cell membranes and mitochondria; lipid peroxidation causes the changes in membrane permeability and disruption in membrane integrity and so disruption of cells (7, 9, 24, 26). 

High concentrations of ROS have important role in pathophysiological damages of human spermatozoa. Therefore; according to studies, oxidative stress is a major cause of infertility in men and in a large percentage of infertile men, a significant increase in activity of ROS levels has been showed in their semen (7, 9, 24, 26, 27). Previous studies concluded that the use of oral antioxidant could improve sperm concentration (28). Overproduction of ROS may induce sperm cell injury through several pathways, motility, and morphology in infertile male (29). Previous studies have shown that the severity of ischemia tissue damage has a direct relationship with duration and degree of testicular torsion (25). 

In our study, administration of troxerutin improves the quality of spermatogenesis and pituitary-gonadal axis hormone against damage caused by ischemia-reperfusion of testis. The results of pituitary-gonadal axis show a significant increase of FSH, LH and testosterone hormone levels in troxerutin group than control group. Luteinizing hormone (LH)/testosterone and follicle-stimulating hormone (FSH) play an important role in the controlling testicular functions (30, 31). LH is released from the pituitary gland, and acts upon the Leydig cells to produce testosterone (30, 31). FSH play essential role in the maturation of germ cells and for the initiation of spermatogenesis. FSHβ or FSH-R knockout mouse models showed that FSH is not critical to maintain fertility (31-33). FSHβ or FSH-R knockout mice have normal spermatogenesis with decreased number of Sertoli cells and sperm but are fertile (31-33). Express FSH, in transgenic hpg mice, can lead to spermatogenesis but not complete germ cell maturation independent of testosterone (31, 34).

Troxerutin known as vitamin P4 is free radical scavenger that protects cells from oxidative stress. Troxerutin can preserve function of cells in the oxidative stress and protect DNA, proteins and lipids from degeneration and apoptosis. Free radicals can reduce the number of sperm and its viability (35). One of the possible mechanisms of troxerutin effect on increasing the number of sperms is due to its antioxidant ability. Vitamin C, a soluble water antioxidant, can protect testis from oxidative stress. It is ROS scavengers that preserve seminiferous tubules, sperm, germ cells and pituitary-gonadal axis from damage-induced by oxidative stress such as hyperthermia, chemotherapy, radiotherapy and inflammation (36-38). Result of this study show that vitamin C only increase level of testosterone in the damage-induced torsion-detorsion testis. Natural and synthetic antioxidants such as melatonin, hydro alcoholic extract of citrus aurantifolia, coriandrum sativum L seed, quercetin, royal jelly and honey can protect testis from damage-induced by ischemia-reperfusion (2, 39-46). 

Jahromi *et al* showed reduction of serum LH concentration after treatment with hydroalcoholic extract of citrus aurantifolia (47). Important compounds of these plants are coumarin and flavonoids (47). The above-mentioned studies are inconsistent with the results of our research; in our study, serum concentration of LH hormone showed a significant increase in receiving troxerutin, a flavonoid, than the control. Quercetin, a flavone, can reduced a significant number of apoptotic germ cells in the model of testicular torsion-detorsion (40). These studies are consistent with the results of our research. Rutin, a bioflavonoid, protect testis from ischemia-reperfusion damage with reduce malondialdehyde levels and increase superoxide dismutase and catalase (48).

Royal jelly preserve spermatogenesis in the mouse induced oxidative injury with oxymetholone (46). Royal jelly can protect seminiferous epithelium and testosterone hormone levels in the treated group (46). These studies are inconsistent with the results of our research. It seems that bioflavonoid family is powerful antioxidants which can play key role as free radical scavenger via activation of xanthine oxidase enzyme that break hypoxanthine to superoxide radicals and acid uric (21). Bioflavonoid with various mechanisms can protect ischemia-reperfusion damage in the tissue. These mechanisms include: prevent neutrophils and mast cells degranulation, scavenger ROS, neutralize nitric oxide, inhibit xanthine oxidase activation, immobilization of leukocyte, and prevent arachidonic acid metabolism, an inflammatory agent. Other effects of bioflavonoid such as quercetin and troxerutin are anti-inflammatory and anti-apoptotic effects in the other tissue (21).

## Conclusion

Taken together troxerutin can protect testis from torsion-detorsion induced injury and preserve seminiferous epithelium, germ cells, sperm and pituitary-gonadal axis from oxidative stress.

## Note

***This article extracted from M.D. Thesis. (Behrouz Rostami)***
